# Experimental demonstration of dual-polarization multiplexed optical phased array empowered by inverse design

**DOI:** 10.1515/nanoph-2025-0148

**Published:** 2025-06-09

**Authors:** Jae-Yong Kim, Seungsoo Lee, Seokjin Hong, Berkay Neseli, Seungyoon Choi, Hyo-Hoon Park, Hamza Kurt

**Affiliations:** The School of Electrical Engineering, 34968 Korea Advanced Institute of Science and Technology (KAIST), Daejeon, Republic of Korea

**Keywords:** silicon photonics, optical phased array, polarization multiplexing, inverse design, optical antenna

## Abstract

This paper presents a dual-polarization multiplexed optical phased array (OPA) implemented on a 220 nm silicon-on-insulator (SOI) platform, enabling continuous beam steering across TE and TM modes. While effectively guiding TE- and TM-polarized light, the proposed OPA on this platform faces the challenge of overcoming the intrinsically high effective refractive index disparity between the two modes upon radiation from the grating antenna. To mitigate this challenge, key OPA components – polarization beam combiner, polarization-independent beam splitter, and index-modulated pixelized grating antenna – were optimized using inverse design methods and integrated, enabling efficient and seamless beam steering across both polarizations. With a 100 nm wavelength tuning range and dual-polarization operation, the fabricated 64-channel OPA achieves a notable longitudinal beam steering range of 34.9° across the TE and TM modes, along with full 2-D beam steering capability. The experimental results confirm the effectiveness of the proposed approach, highlighting its potential for advancing the next-generation LiDAR and optical wireless communication systems.

## 1 Introduction

Optical phased arrays (OPAs) have been extensively studied for their solid-state beam steering capabilities, establishing them as a key technology in applications such as light detection and ranging (LiDAR) imaging [1], [2], [3], optical wireless communication (OWC) [4], [5], [6], digital holography [7], and bio-sensing [8]. In particular, significant research has been implemented on the silicon-on-insulator (SOI) platform, due to its advantages, including CMOS compatibility, low power consumption, compact footprint, and high integration density [9], [10], [11], [12], [13], [14], [15], [16]. Leveraging their capabilities to accommodate diverse passive and active components in the SOI platform, OPAs have driven the exploration of various beam steering strategies, while expanding the field-of-view (FOV) in both transverse and longitudinal directions, meeting the stringent-performance requirements of LiDAR and optical wireless communication systems. In conventional 1-D OPA, transversal beam steering is commonly achieved through active phase control in each channel, adopting either thermo-optic (TO) or electro-optic (EO) tuning mechanisms [13], [17], or passively through employing optical path length difference and wavelength tuning approaches [18], [19]. To further increase the FOV in the transverse directions, submicron-pitch [9], and aperiodic antenna arrays [20] have demonstrated the ability to achieve beam steering beyond 120° with active phase control.

Longitudinal beam steering is typically achieved through effective refractive index tuning or wavelength tuning in a grating antenna array, in accordance with the Bragg condition. However, conventional designs often limit the longitudinal FOV to approximately 10°–15° [16], [21]. To extend this range, one recent approach with polarization multiplexing techniques in OPAs has been proposed as a promising solution [22], [23], [24], [25], [26]. This method necessitates the integration of polarization-independent components like beam splitters, or polarization-selective components like polarization beam splitters/rotators, and grating antennas capable of continuous beam steering across both TE and TM modes. Although this method increases the circuit complexity, it theoretically enables a steering range of 28.2° with a wavelength tuning range of 100 nm [22]. Furthermore, incorporating a bidirectional structure can extend the steering range even further, up to 54.5°, as calculated [23].

Despite this potential, this approach has been primarily explored on thicker SOI platforms, such as 340 nm and 290 nm, where the smaller index gap between TE and TM modes in antenna facilitates continuous beam steering [22], [23], [25]. These studies on thicker platforms have provided theoretical and structural insights; however, experimental validation has yet to be demonstrated in most cases. In case of the widely adopted 220 nm SOI platform, achieving seamless beam steering across both polarizations involves considerable design sensitivity, primarily due to the significant effective refractive index difference between the TE and TM modes in grating antennas [22]. Although research efforts to implement polarization-multiplexed OPAs on this platform, one study involving a 4-channel configuration with a conventional grating antenna structure revealed beam steering discontinuities between TE and TM modes, with an angular gap of nearly 40° [24]. To address this, a method employing physically separated dual antennas for TE and TM modes was proposed, enabling continuous beam steering in a 16-channel OPA [26]. While effective, this design choice poses potential challenges such as crosstalk between the adjacent antennas and constraints in reducing antenna pitch to expand the transverse beam steering angle. To overcome these issues, inverse design methods that induce index modulation within grating antennas offer a promising solution for achieving continuous beam steering across both TE and TM modes within a single structure on a 220 nm SOI platform. Additionally, optimizing other key components, including polarization beam combiners and polarization-independent beam splitters, is also essential to support polarization handling within the scaled-up integrated OPA system.

In this paper, we designed a 64-channel polarization multiplexing OPA on a 220 nm SOI platform and experimentally demonstrated the continuous longitudinal beam steering with a 100 nm wavelength tuning range and dual-polarization operations. By integrating optimally designed components through inverse design techniques, including a polarization beam combiner, polarization-independent beam splitters, and index-modulated grating antennas, the OPA enabled seamless longitudinal beam steering with a steering efficiency of 0.349°/nm across both TE and TM modes, while achieving the 2-D beam steering with the beam steering angles of 41°/34.9°, along with corresponding divergence angle of 0.86°/1.38° for TM mode beam and 0.82°/2.48° for TE mode beam, on average.

## 2 Design and simulation

**Figure 1:**
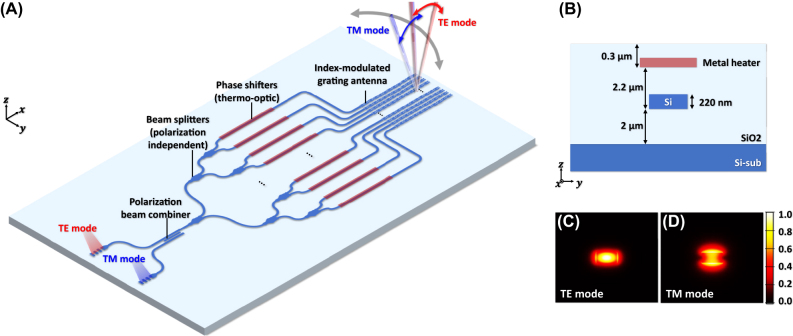
Overall structure of the proposed dual-polarization multiplexed OPA system implemented on a 220 nm SOI platform. (A) Schematic of the proposed polarization multiplexing optical phased array (OPA) along with its building blocks. (B) Layer stack of the 220 nm silicon-on-insulator (SOI) platform. (C) Cross-sectional view of TE mode confinement profile and (D) TM mode confinement profile in a waveguide with a 400 nm width.

The architecture of the proposed 64-channel polarization multiplexing OPA is illustrated in Figure 1A, which is implemented on a commonly used 220 nm SOI platform. The OPA comprises a TE grating coupler for TE-polarized input light, a TM grating coupler for TM-polarized input light, a polarization beam combiner, a six-stage polarization-independent beam splitter tree, 64-channel TO phase shifters, and index-modulated grating antenna array. Polarized light as either TE or TM mode by an external polarization controller is coupled into the corresponding TE or TM grating coupler. The light then passes through the polarization beam combiner and is guided along a single waveguide. The guided light is subsequently distributed evenly into 64 channels by six stage polarization-independent beam splitter tree, ensuring uniform optical power distribution. Then, the light in each channel is phase-modulated by a 400 µm-long thermo-optic phase shifter before propagating through phase-feeding lines toward the grating antennas. Finally, the phase-modulated TE or TM polarized light is emitted through the index-modulated grating antennas with its longitudinal radiation angle determined by the wavelength and polarization state of light.

In the transverse direction, OPA beam steering is achieved through thermo-optic tuning of individual phase shifters. More critically, in the longitudinal direction, OPA beam steering is controlled by wavelength tuning within the target range of 1,260 nm–1,360 nm, effectively doubling the steering range by leveraging both TE and TM modes. A key requirement here is ensuring continuous angular coverage across both polarization states. To realize this, it is essential to optimize all components responsible for guiding the two polarization states toward the grating antennas, minimizing propagation loss within the targeted wavelength tuning range. Furthermore, index-modulated grating antennas are employed to overcome the beam steering discontinuity caused by the large effective refractive index gap between TE and TM modes in conventional strip waveguides on the 220 nm SOI platform, thereby enabling continuous beam steering with a minimal wavelength tuning range.

Sections 2.1 to 2.3 present a comprehensive explanation of the core building blocks of the polarization multiplexing OPA, detailing the optimized designs of the polarization beam combiner, polarization-independent beam splitter, and index-modulated grating antenna using inverse-design methodologies.

### 2.1 Polarization beam combiner

**Figure 2:**
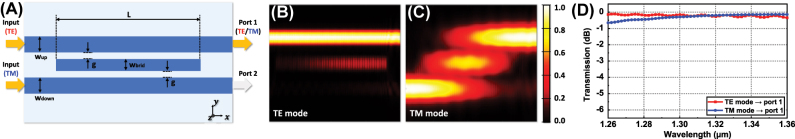
Design and simulation results of the polarization beam combiner. (A) Schematic of the polarization beam combiner, including structural parameters such as waveguide dimensions and coupling characteristics. Simulated results of the optimized polarization beam combiner: H-field propagation in the polarization beam combiner for (B) TE-polarized and (C) TM-polarized input light. (D) Calculated transmission efficiency into port 1 for TE and TM polarizations.

A polarization beam combiner is required for effective multiplexing of the light coupled from TE and TM grating couplers into a single-strip waveguide. It is intended to efficiently merge the light from separate waveguides supporting distinct polarization states into a single waveguide while ensuring minimal insertion loss across our targeted wavelength range of 1,260–1,360 nm. The schematic of the polarization beam combiner in the OPA is illustrated in Figure 2A. The structure consists of two strip waveguides and a bridge waveguide within the coupling region, mirroring the overall configuration of a directional coupler-based polarization beam splitter [27]. TE- and TM-polarized light are individually introduced from the upper and lower strip waveguides, respectively, passing through the polarization beam combiner and ultimately exiting through port 1. The structural parameters include *W*_up_ and *W*_down_, which correspond to the widths of the upper and lower strip waveguides, respectively, and *W*_brid_ denoting the width of the bridge waveguide. The gap g represents the separation between the upper (or lower) waveguide and the bridge waveguide, while the coupling length between adjacent waveguides is denoted as *L*. The design parameters were initially determined using coupled mode theory (CMT) to satisfy the phase-matching condition and the super-mode approach for the TM mode [27], [28], [29], followed by fine-tuning with particle swarm optimization (PSO) algorithm to achieve high transmission in port 1 for both TE and TM mode. The optimized widths of the upper $\left(W_{\text{up}}\right)$ and lower $\left(W_{\text{down}}\right)$ strip waveguides are both set to 400 nm, while the bridge waveguide has a width *W*_*brid*_ of 425 nm. The gap *g* between the upper strip and the bridge, as well as between the lower strip and the bridge, is set at 200 nm, with a coupling length *L* of 16 µm for both.

The simulated propagation characteristics of TE- and TM-polarized light in the optimized polarization beam combiner are shown in Figure 2B and C. TE light propagates directly to port 1, while TM light is coupled into port 1 with the aid of the bridge waveguide. The corresponding transmission spectra in Figure 2D show that at a wavelength of 1,310 nm, the transmission at port 1 is −0.2 dB for TE mode and −0.24 dB for TM mode. Across the wavelength range from 1,260 nm to 1,360 nm, the minimum transmission is −0.34 dB for TE and −0.65 dB for TM, respectively, indicating sufficient performances for polarization multiplexing in the proposed scheme.

### 2.2 Polarization-independent beam splitter

To enable polarization multiplexing in OPA, polarization-independent beam splitters are crucial in dividing a single input light into multiple channels while accommodating both TE and TM polarizations. A fundamental requirement of these beam splitters is that TE- or TM-polarized input light should be evenly distributed into two output ports while minimizing the propagation losses within the target wavelength range of 1,260–1,360 nm.

**Figure 3:**
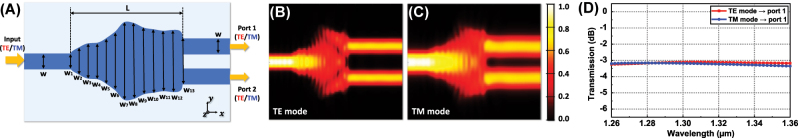
Design and simulation results of the polarization-independent beam splitter. (A) Schematic of the polarization-independent beam splitter with structural parameters, including the interpolated width variations and splitter length. Simulated results of the optimized polarization-independent beam splitter: H-field propagation in the beam splitter for (B) TE and (C) TM input polarizations. (D) Calculated transmission into port 1 for TE and TM polarizations (port 2 exhibits identical transmission due to symmetry in the *y*-direction).

To achieve this, we designed the polarization-independent beam splitter using an inverse design approach, ensuring a symmetric structure along the *y*-axis, as illustrated in Figure 3A. Adopting a design method similar to that used in the previous work on a 1 × 4 beam splitter device [30], we defined 13 parameters with varying widths along the length direction and applied spline interpolation. TE- or TM-polarized light, originating from a waveguide with a width w of 400 nm propagates through the width-interpolated power splitter and is evenly distributed into two output ports retaining the same width w of 400 nm as the input waveguide. To mitigate evanescent coupling between the ports, a minimum separation constraint of 1.1 µm was imposed at the final width $\left(W_{13}\right)$ . The parametric optimization was then carried out using the PSO algorithm for 13 distinct widths $\left(W_{1}-W_{13}\right)$ and splitter length (*L*), with the Figure-of-Merit (FoM) defined to ensure that the transmission of TE- and TM-polarized light from the input port to ports 1 and 2 approaches 0.5, respectively, within the given wavelength range of 1,260–1,360 nm. The optimized parameters are listed in Table 1.

**Table 1:** Optimized parameters in polarization-independent beam splitter (unit: µm).

W_1_	W_2_	W_3_	W_4_	W_5_	W_6_	W_7_
0.4	0.54	0.66	0.67	1.01	1.39	1.78
**W**_**8**_	**W**_**9**_	**W**_**10**_	**W**_**11**_	**W**_**12**_	**W**_**13**_	**L**
1.66	1.55	1.46	1.39	1.5	1.1	3.4

The simulated field propagation in the optimized beam splitter is shown in Figure 3B and C for TE- and TM-polarized input light, respectively. The results confirm that the beam is evenly distributed into port 1 and port 2, regardless of the polarization. The corresponding transmission spectra in Figure 3D show that at 1,310 nm, TE mode transmits −3.1 dB and TM mode −3.2 dB to port 1, with identical power levels reaching port 2 due to the symmetric configuration. The minimum transmission across the given wavelength range is −3.24 dB for TE and −3.35 dB for TM, ensuring efficient beam splitting with minimal optical loss.

### 2.3 Index-modulated grating antenna

At the final stage, after the phase modulation by the thermo-optic phase shifter in each channel, the antennas should emit the beams in a well-controlled manner to align with the designated wavelength and polarization state. To achieve continuous beam steering in the longitudinal direction, the emission angles covered by TE- and TM-polarized light within the given wavelength ranges should be designed to exhibit slight overlap with effective refractive index engineering. Simultaneously, it is necessary to extend the effective propagation length in the antenna, which is the preferred characteristic to minimize the beam divergence angle, while increasing the upward emission for both polarization states. Given these conditions, the design considerably increases complexity, making it difficult to rely solely on conventional structures, such as fishbone-shaped [31], shallow-etched [12], or cladding-modulated grating antennas [32].

Especially, it should be noted that the effective refractive index difference between the TE and TM modes in a standard strip waveguide is notably larger in the commonly used 220 nm SOI platform, compared to the 340 nm-thick SOI platform [22]. For a typical waveguide width of 400 nm in the O-band, this difference is approximately 0.5. The beam radiation angle θ in a grating antenna is determined by the following equation:

(1) $$\theta =\sin^{-1}\left(\frac{n_{\text{eff}}}{n_{c}}-\frac{\lambda_{0}}{n_{c}\Lambda }\right),$$

where *n*_eff_ is the effective refractive index of the guided light, *n*_*c*_ is the background index (assumed to be 1 for air), *λ*_0_ is the free-space wavelength, and Λ is the grating period. For instance, in a 400 nm-wide waveguide, the TE mode exhibits an effective refractive index of 2.57, while the TM mode has an index of 2.08 at 1,310 nm wavelength. When designing a grating antenna with a period of 600 nm, the calculated radiation angles are 22.8° for the TE mode, and −5.9° for the TM mode at the wavelength of 1,310 nm, resulting in an angular difference of about 28.7°. This substantial difference makes it require a wide wavelength tuning range. Given that typical beam steering efficiency is 0.1–0.15°/nm, compensating for this discrepancy would require a range of nearly 200 nm, which poses practical challenges. Due to these intrinsic limitations, conventional grating antennas are inadequate for effectively addressing the issue. To achieve continuous beam steering across both polarizations over a practical wavelength tuning range within 100 nm, the index gap should be maintained around 0.2 [22]. To overcome this, we implemented an inverse design optimization with a binary PSO algorithm to generate a pixelized, index-modulated grating antenna, thereby optimizing the beam steering characteristics to align with the intended performance objectives.

**Figure 4:**
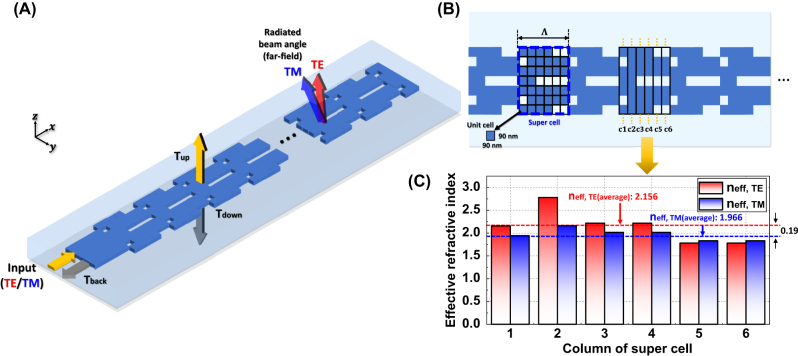
Design and calculated index profiles of the index-modulated grating antenna. (A) Schematic of the proposed index-modulated grating antenna and (B) structural parameters, including supercells composed of 42-unit cells. (C) Calculated effective refractive indices for TE and TM modes in each column of the supercell at a wavelength of 1,310 nm.

As depicted in Figure 4A and B, an index-modulated antenna was designed and optimized with a periodically repeating supercell structure. Each supercell comprises 42-unit cells, which were optimized using a binary PSO algorithm to determine the material composition as either silicon (Si) or silicon dioxide (SiO_2_) while maintaining structural symmetry along the *y*-axis to facilitate the optimization process. The unit cell dimensions were set at 90 nm × 90 nm, considering the minimum feature size feasible for electron beam lithography fabrication, and the corresponding grating period was 540 nm. To achieve several performance objectives – including continuous beam steering across both TE and TM polarizations with maximized upward directivity, and propagation length – a multifunctional FoM was formulated, as follows:

(2) $$\begin{array}{ll} \text{FoM}\left(\min\;.\right) & =a\left(\theta_{TE,\lambda_{\min}}-\theta_{TM,\lambda_{\max}}\right)\\ & \;+b\left(\alpha -\frac{P_{\text{up}}}{P_{\text{up}}+P_{\text{down}}+P_{\text{back}}}\right)+c\left(\beta -\frac{P_{\text{remain}}}{P_{\text{in}}}\right), \end{array}$$

where the first term ensures continuity in the radiation angles between TE and TM mode with $\theta_{TE,\lambda_{\min}}$ representing the radiation angle at the targeted minimum wavelength with TE mode and $\theta_{TM,\lambda_{\max}}$ denoting the radiation angle at the targeted maximum wavelength with TM mode. The second term is for maximizing the upward directivity where *P*_up_, *P*_down_, and *P*_back_ represent the upward emission power, downward emission power, and backward reflected power, respectively, while *α* defines the target directivity. The third term optimizes propagation length. Here, within a designated simulation region containing N supercells, *P*_in_ represents the input power, while *P*_remain_ corresponds to the power remaining after propagating through the N supercells, with *β* specifying the desired fraction of the remaining power. Each term is weighted by the constant *a*, *b*, *c*, balancing these objectives. By appropriately adjusting these weights through iterative refinement, an optimized design was obtained shown in Figure 4B, effectively reducing the gap between *n*_eff,TE_ and *n*_eff,TM_ in Eq. (1), while simultaneously enhancing the directivity and propagation length.

Figure 4C shows the effective refractive indices of the TE and TM modes propagating through columns 1 to 6 of the optimized supercell at the wavelength of 1,310 nm. As the effective refractive indices of *n*_eff,TE_ and *n*_eff,TM_ can be approximated by averaging the values over each grating cell column, they are calculated as 2.156 and 1.966, respectively, resulting in a difference of 0.19. This indicates that the inverse design optimization effectively reduced the intrinsic index gap in the grating antenna region.

**Figure 5:**
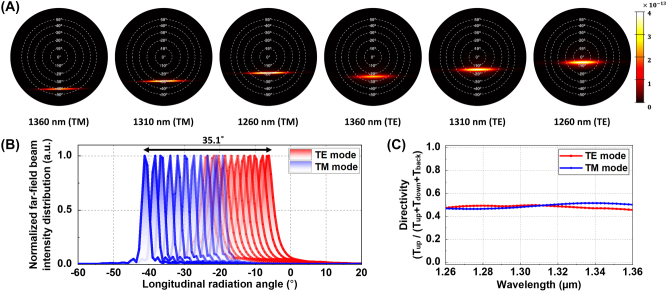
Simulation results of beam steering characteristics of the optimized index-modulated grating antenna. (A) Simulated far-field beam profiles from the optimized grating antenna, illustrating the variation in longitudinal radiation angles for TM mode at wavelengths of 1,360 nm, 1,310 nm, and 1,260 nm, and for TE mode at wavelengths of 1,360 nm, 1,310 nm, and 1,260 nm. (B) Simulated results in the optimized grating antenna, presenting the normalized far-field beam intensity distribution along the longitudinal direction for TE and TM modes across wavelengths ranging from 1,360 nm to 1,260 nm in 10 nm decrements and (C) the directivity variation from 1,260 nm to 1,360 nm for TE mode and TM mode, respectively.

Figure 5A presents the far-field beam profiles emitted from an optimized single grating antenna when subjected to TE- and TM-mode inputs while varying the wavelength. A slight angular overlap is observed between TM mode at 1,260 nm and TE mode at 1,360 nm, suggesting the potential for continuous beam steering in the longitudinal direction. The far-field beam intensity distribution across the wavelength range of 1,360 nm–1,260 nm in 10 nm decrements is shown in Figure 5B. Within this spectral window, the TM-mode exhibits a tunable range from −41.2° to −19.1° with the corresponding tuning efficiency of 0.221°, while the TE-mode extends from −23.3° to −6.1° with the tuning efficiency of 0.172°. This results in an overlapping region of 4.2° between two polarization states. Consequently, this combined approach of 100 nm wavelength tuning and TE-/TM-polarization switching enables continuous beam steering over a total angular range of 35.1°, indicating a beam steering efficiency of 0.351°/nm from the numerical simulations. For directivity, as shown in Figure 5C, the TE mode exhibits higher efficiency, with values ranging from 45.7 % to 49.3 %. In contrast, the TM mode exhibits a slightly higher directivity ranging from 46.5 % to 51.6 %. These results indicate that the overall performance remains adequate for the proposed OPA operation, supporting continuous beam steering and validating the effectiveness of the multifunctional optimization.

## 3 Fabrication and experimental verification

### 3.1 Fabrication of OPA

**Figure 6:**
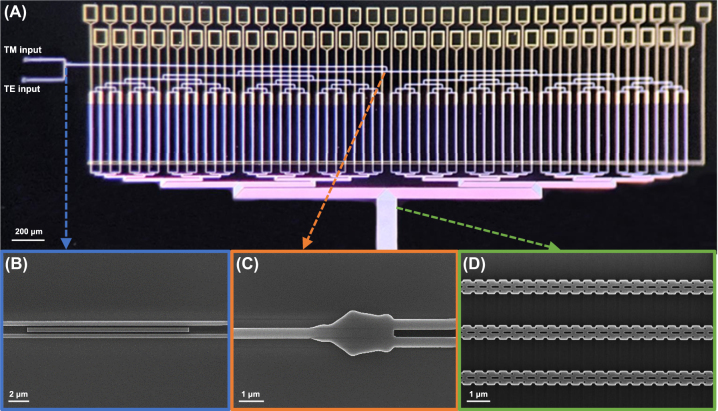
Images of the fabricated OPA chip and its main components. (A) Optical microscope image of the fabricated OPA chip. SEM images of the main components in the OPA; (B) polarization beam combiner, (C) polarization-independent beam splitter, and (D) index-modulated grating antennas.

We fabricated a 64-channel OPA chip using the 220 nm SOI multiproject wafer run provided by Applied Nanotools (ANT) foundry. The chip features a layered structure consisting of a 2 µm oxide layer, a 220 nm silicon layer above it, and a 2.5 µm oxide cladding on top. TiW heater and Au routing layer were incorporated to enable thermo-optic tuning in phase shifters. The foundry’s 100 keV electron beam lithography capabilities, which allow for device patterning of small features down to 60 nm size, enable to fabrication of all OPA components, including the pixelized index-modulated grating antennas. A uniform pitch of 2 μm was employed in the antenna array configuration to ensure negligible crosstalk between adjacent channels, while supporting a sufficient transversal beam steering range over 40°. The overall footprint of the OPA chip including the electrical pads was 4.3 mm×1.5 mm. The fabricated OPA chip with the main components is shown in Figure 6A–D. After the fabrication process, the chip was packaged in a chip-on-board configuration by electrically wire-bonding it to metal PCB pads. Parallel testing was conducted on each component using test patterns.

### 3.2 Experimental verification

**Figure 7:**
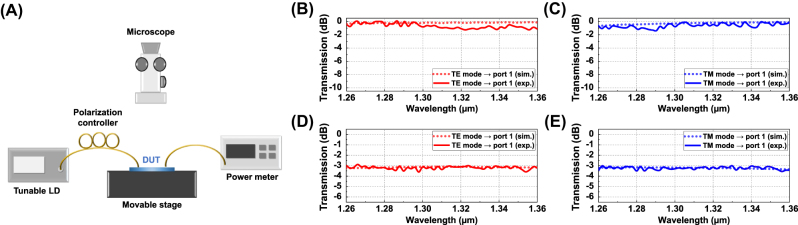
Experimental setup and characterization results of individual components. (A) Experimental setup for measuring the individual devices. Measurement results of the polarization beam combiner for (B) TE mode and (C) TM mode, and the polarization-independent beam splitter for (D) TE mode and (E) TM mode, all overlaid with the corresponding simulations results.

The experimental setup for individual device characterization, as shown in Figure 7A, was configured as follows. A tunable laser source (TSL-550), supporting wavelengths from 1,260 nm to 1,360 nm, was connected to a polarization controller to selectively adjust the polarization state to either TE or TM mode. Then, the polarized light was vertically coupled into the OPA chip via the corresponding input TE or TM grating coupler, propagating through the test pattern structures. The output light was then collected from the output grating coupler, coupled into an optical fiber, and directed to a power meter for measurement. The spectral response of each device was obtained by normalizing it against a reference pattern consisting of a symmetric grating coupler pair with an identical optical path length. For reference, fully etched focused grating couplers were used for TE and TM mode coupling, yielding coupling losses of −10.4 dB, and −8.2 dB, respectively, with an 8° fiber coupling angle at 1,310 nm.

Figure 7B–E presents the measured transmissions for TE and TM modes in both the polarization beam combiner device and the polarization-independent beam splitter, overlaid with the corresponding simulation results. The polarization beam combiner achieved an averaged excess loss of 0.67 dB for the TE mode and 0.64 dB for the TM mode within the given range, as shown in Figure 7B and C, demonstrating good agreement with the optimized design. Likewise, the polarization beam splitter exhibited an averaged excess loss of 0.17 dB for the TE mode and 0.22 dB for the TM mode as shown in Figure 7D and E, aligning well with the simulated results. While some wavelength points showed higher losses up to 1.27 dB (TE) / 1.38 dB (TM) in beam combiner and 0.58 dB (TE) / 0.53 dB (TM) per port in beam splitter, such localized variations are likely due to fabrication and measurement fluctuations. Nonetheless, the overall performance of both components appears reasonable to support in polarization routing within the integrated OPA system.

**Figure 8:**
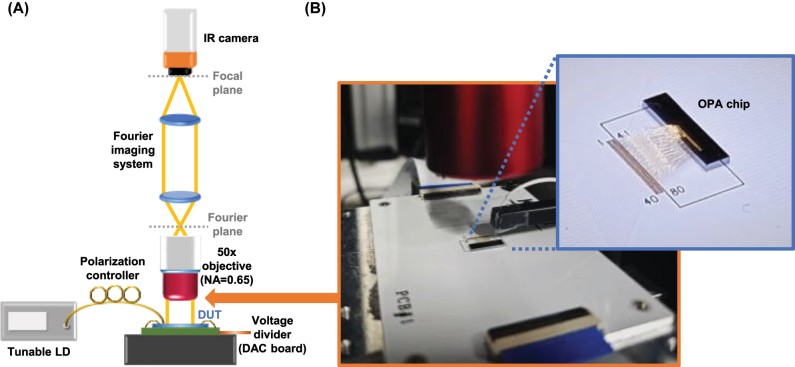
Experimental setup and packaged OPA module for far-field beam pattern measurements. (A) Schematic of the experimental setup for characterizing the radiation patterns of the OPA. (b) Packaged OPA with electrical wire bonding to a printed circuit board (PCB).

In addition, the far-field beam patterns of the packaged OPA chip were characterized using an experimental setup shown in Figure 8A and B. As with the test pattern measurement, a tunable laser diode (LD) with a wavelength range of 1,260–1,360 nm, along with a polarization controller, was used to selectively couple TE- or TM-polarized light into the corresponding grating coupler. The far-field patterns emitted from the OPA were captured using a Fourier imaging system, which included an high-NA objective lens, followed by two external lenses – one for collimating the light to define the Fourier plane and the other for projecting the image onto an IR camera, as illustrated in Figure 8A. A digital-to-analog converter (DAC) board was employed to distribute voltages to 64 electrodes in the OPA module. The DAC board was connected to a customized LabVIEW-based control system on the PC, facilitating the voltage control for each phase shifter as in our previous work [33]. For reference, the thermo-optic efficiency of a 400-µm phase shifter in the OPA achieved a π-phase shift with a power consumption of 18 mW for TE mode and 18.9 mW for the TM mode.

**Figure 9:**
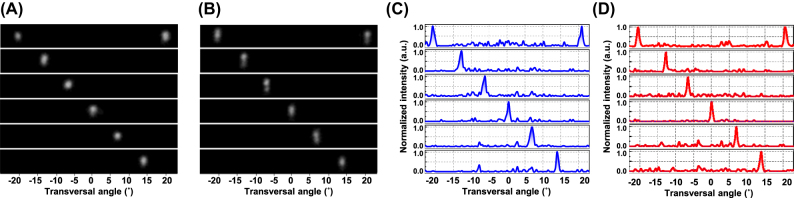
Representative measured far-field beam patterns of OPA demonstrating transversal beam steering at a wavelength of 1,310 nm for 7 different directions in (A) TM mode and (B) TE mode. The corresponding normalized beam intensity distributions for (C) TM mode and (D) TE mode.

To evaluate the OPA beam steering performance along the transversal direction, far-field measurements were carried out at a wavelength of 1,310 nm under TE- and TM-mode conditions, respectively. At this wavelength, the TM mode beams were positioned at −30.2° and the TE mode beams at −13.9° in the vertical direction. Within the theoretical steering range of 41°, phase calibration was carried out for 7 representative emission angles per polarization, including the maximum angle where dual main beams, typically observed in uniform-array structures. For each angle, the phase settings of all 64 thermo-optic shifters were individually optimized using a hill-climbing algorithm. The corresponding far-field radiation patterns are shown in Figure 9A and B for the TM and TE modes, respectively, while the normalized intensity distributions are presented in Figure 9C and D. The average side-mode suppression ratios (SMSRs) were measured to be 7.0 dB for the TM mode and 7.3 dB for the TE mode with minimum values of 5.0 dB (TM) and 6.1 dB (TE), respectively. These modest values were attributed to grating lobe noise during phase calibration, which becomes more pronounced as the radiation angle increases, as well as to the limitations of the local optimization algorithm. Improved SMSR performance is expected through the suppression of grating lobes employing an aperiodic antenna arrangement, along with more advanced calibration methods such as gradient descent or global optimization algorithms. Nevertheless, robust and consistent beam steering was achieved in the transversal direction for both polarizations.

**Figure 10:**
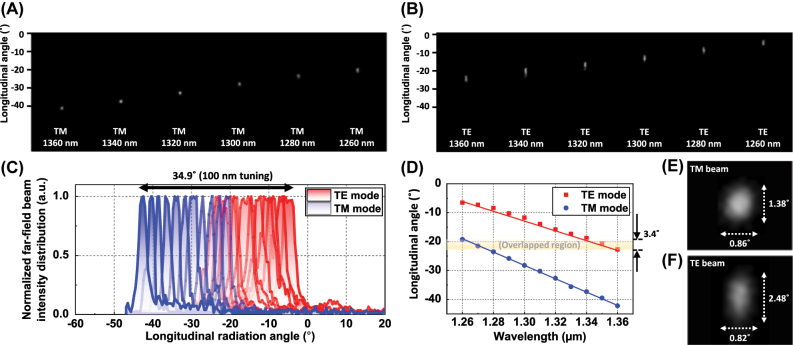
Measured OPA far-field beam steering in the longitudinal direction with wavelength tuning from 1,260 nm to 1,360 nm with 20 nm wavelength steps in (A) TM mode and (B) TE mode. (C) Normalized intensity distributions of far-field beams for TE and TM modes and (D) longitudinal beam radiation angles for TE and TM modes over a 100 nm wavelength tuning with 10 nm wavelength intervals. The averaged divergence angles of the radiated (E) TM beam and (F) TE beam.

The longitudinal beam steering characteristics of the OPA were examined under wavelength tuning from 1,360 nm to 1,260 nm in 20 nm decrements for the TM and TE modes, respectively. As in the transversal case, phase calibration was individually performed at each wavelength step and polarization, with the same transversal emission angles. Minor thermally induced phase drifts occurring at each wavelength step were also compensated accordingly. Under this procedure, the TM-mode beam was initially directed to −41.4° at 1,360 nm and gradually steered to −19.4° at 1,260 nm, covering a total angular shift of 22°, as shown in Figure 10A. Likewise, the TE mode beam moves from −22.7° to −6.5° over the same tuning range, as presented in Figure 10B. The experimentally measured steering efficiencies of 0.22°/nm for TM and 0.162°/nm for TE closely align with the theoretical values of 0.221°/nm and 0.172°/nm, respectively. Including the overlapped region of 3.4° between both polarizations, as shown in Figure 10C and D, the total longitudinal beam steering range extends to 34.9° across the 100 nm tuning span, corresponding to a steering efficiency of 0.349°/nm. While slight irregularities are observed in the TE-mode beam profiles at certain wavelengths, as seen in Figure 10C, likely caused by measurement deviations, the overall steering behavior, as shown in Figure 10D, remains consistent with the expected trend. With the exception of the antenna emission efficiency, which could not be characterized due to radiation in the negative direction and experimental constraints, the remaining findings showed strong agreement with the simulations. This indicates that the proposed device performs as designed, achieving the intended and enhanced beam-steering characteristics in the longitudinal direction, as well.

With average beam divergence angles of 0.86°/1.38° for the TM mode and 0.82°/2.48° for the TE mode, a notable longitudinal divergence difference of 1.1° was observed between the two polarizations, as shown in Figure 10E and F. This non-negligible divergence angle and the polarization-dependent discrepancy stem from noticeable yet slightly different grating strengths in the optimized antenna. To mitigate this, the effective index difference between the two halves of the supercell be minimized and balanced for each polarization mode. It is also expected that further enhancement in divergence angle reduction can be achieved by employing additional etching steps to create a greater diversity of index modulation variations. Furthermore, our preliminary results indicate that the proposed antenna design approach can be extendable to the widely used C-band region, enabling further performance improvements through appropriate structural optimization.

## 4 Conclusions

In this study, a dual-polarization multiplexed OPA capable of continuous beam steering across the two polarizations has been designed and experimentally demonstrated. Notably, the significance of this work lies in the successful implementation of dual-polarization OPA capable of continuous beam steering, enabled by a pixelated grating antenna optimized through inverse design techniques. In conjunction, polarization-handling components including the polarization beam combiner and polarization-independent beam splitter were also designed to support dual-polarization routing, maintaining full compatibility with the 220 nm SOI platform. With wavelength tuning range of 100 nm and dual-polarizations, we achieved continuous beam steering angle of 34.9° in the longitudinal direction, which is approximately 2–3 times wider than that of conventional OPAs within the same tuning range. By addressing the key challenges in polarization-multiplexed beam steering in OPA through the seamless integration of inverse-designed photonic components, this work is expected to pave the way for next-generation OPA systems, including applications in LiDAR, optical wireless communication, and other emerging fields.
